# Association of thrombogenicity indices with perioperative cardiovascular events after non-cardiac surgery: a prespecified analysis of the PANDA study

**DOI:** 10.1007/s11239-026-03299-z

**Published:** 2026-05-18

**Authors:** Hendrianus Hendrianus, Jong-Hwa Ahn, Min-Gyu Kang, Kye-Hwan Kim, Jin-Sin Koh, Sang-Wook Kim, Jin-Yong Hwang, Udaya S. Tantry, Paul A. Gurbel, Jeong-Rang Park, Young-Hoon Jeong

**Affiliations:** 1 Division of Cardiology, Chung-Ang University Gwangmyeong Hospital, Gwangmyeong, South Korea; 2 Department of Internal Medicine, Chung-Ang University College of Medicine, Seoul, South Korea; 3 Division of Cardiology, Natuna General Hospital, Natuna, Indonesia; 4 Cardiovascular Center, Gyeongsang National University Changwon Hospital, Changwon, South Korea; 5 Department of Internal Medicine, Gyeongsang National University School of Medicine, Jinju, South Korea; 6 Division of Cardiology, Gyeongsang National University Hospital, Jinju, South Korea; 7 Sinai Center for Thrombosis Research and Drug Development, Sinai Hospital of Baltimore, Maryland, USA; 8 CAU Thrombosis and Biomarker Center, Chung-Ang University Gwangmyeong Hospital, Gwangmyeong, South Korea

**Keywords:** Surgery, Perioperative risk, Revised Cardiac Risk Index, Thrombogenicity, Coronary artery disease, Cardiovascular events

## Abstract

Traditional clinical risk models, such as Revised Cardiac Risk Index (RCRI), have limited predictive value for estimating postoperative cardiovascular complications following non-cardiac surgery. This analysis aimed to evaluate prognostic value of thrombogenicity profiles and coronary anatomy for cardiovascular events in patients undergoing non-cardiac surgery. In a prospective cohort of 120 patients who underwent intermediate-to-high risk surgery, thrombogenicity profiles were assessed using thromboelastography (TEG®) and conventional hemostatic measurements before surgery. Coronary artery disease (CAD) was preoperatively defined as presence of significant stenosis (≥ 50% luminal narrowing) on coronary computed tomography angiography (CCTA). Postoperative cardiovascular events were defined as cardiovascular death, non-fatal myocardial infarction, myocardial injury, pulmonary edema, non-fatal stroke, and systemic embolism within 30 days after surgery. Sixteen patients (13.3%) experienced cardiovascular events. In multivariable analysis, presence of CAD (odds ratio [OR]: 5.11; 95% confidence interval [CI]: 1.49–17.53; *P* = 0.009), D-dimer (per 1-μg/mL increase: OR: 1.22; 95% CI: 1.02–1.47; *P* = 0.030), and platelet–fibrin clot strength (PFCS) measured by TEG® (per 1-mm increase: OR: 1.10; 95% CI: 1.01–1.20; *P* = 0.027) were independently associated with cardiovascular events. Discrimination of cardiovascular event risk improved progressively with the sequential addition of the following risk stratification models: RCRI alone, RCRI + CCTA, and RCRI + CCTA + thrombogenicity profiles (C-index: 0.660 vs. 0.731 vs. 0.803). Cardiovascular event rates increased with greater risk burden, ranging from 4.2% in patients with no risk components to 77.8% in those with all components present. Integrating thrombogenicity assessment and CCTA with traditional clinical risk models may improve perioperative risk stratification and help guide tailored preventive strategies for patients undergoing non-cardiac surgery.

*Clinical trial registration*. URL: http://www.clinicaltrials.gov. Unique identifier: NCT02250963.

**Graphical Abstract:**

Sequential integration of thrombogenicity profiles and coronary anatomy assessed by CCTA improves perioperative cardiovascular risk prediction beyond clinical risk stratification alone in patients undergoing non-cardiac surgery. CAD = coronary artery disease; CCTA = coronary computed tomography angiography; CI = confidence interval; CV = cardiovascular; MI = myocardial infarction; MINS = myocardial injury in non-cardiac surgery; PFCS = platelet–fibrin clot strength; RCRI = Revised Cardiac Risk Index; TEG® = thromboelastography.

**Figure:**
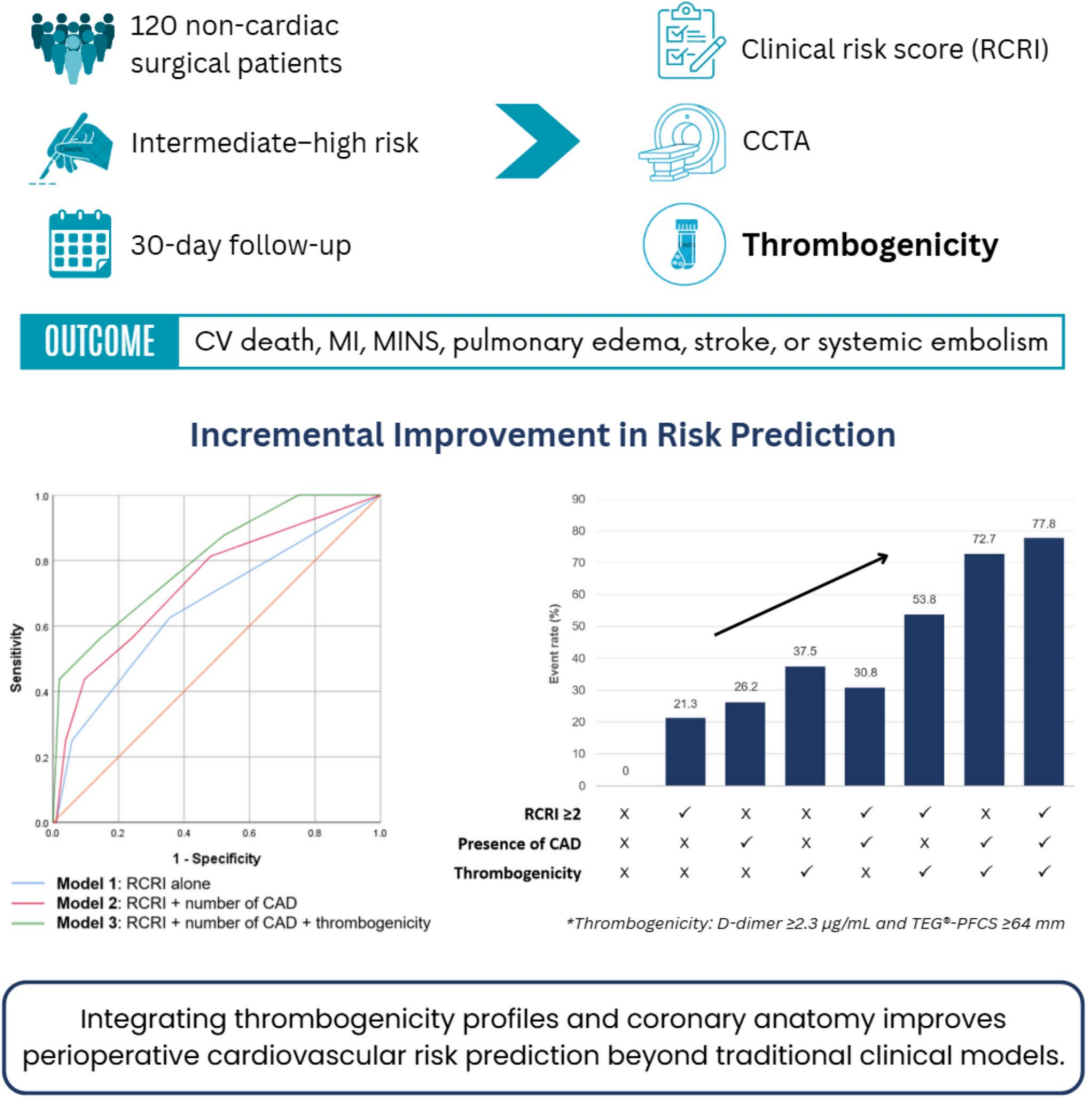

**Supplementary Information:**

The online version contains supplementary material available at 10.1007/s11239-026-03299-z.

## Introduction

Cardiovascular complications remain a leading cause of perioperative morbidity and mortality in patients undergoing non-cardiac surgery, particularly in those with known or occult coronary artery disease (CAD) [1, 2]. Events such as myocardial infarction (MI), myocardial injury after non-cardiac surgery (MINS), stroke, and cardiovascular death contribute substantially to this risk. As surgical populations age and comorbidity burden increases, accurate preoperative cardiovascular risk stratification is essential to optimize management and improve outcomes [3].

Conventional tools, including the Revised Cardiac Risk Index (RCRI), provide a practical framework for risk assessment but have limited accuracy, particularly in identifying patients with subclinical or anatomically significant CAD [4]. Coronary computed tomography angiography (CCTA) enables direct visualization of coronary atherosclerosis and offers incremental prognostic value, especially in intermediate-risk surgical populations [5, 6].

However, perioperative cardiovascular risk is not determined by anatomical disease burden alone. Dynamic processes such as inflammation, endothelial dysfunction, and a prothrombotic state also play a central role [7]. These mechanisms can be captured by circulating and functional biomarkers. D-dimer reflects fibrinolysis and systemic thrombotic activity and has been consistently associated with adverse outcomes across clinical settings [8, 9]. In parallel, thromboelastography (TEG®) provides a global assessment of clot formation, strength, and fibrinolysis [10, 11]; its platelet–fibrin clot strength (PFCS) parameter has been linked to atherothrombotic risk in cardiovascular and surgical populations [10, 12, 13].

Despite these advances, the incremental prognostic value of integrating anatomical imaging with thrombogenicity profiles in the perioperative setting remains unclear. We therefore conducted this prospective substudy to evaluate whether combining CCTA-derived CAD assessment with thrombogenicity markers improves prediction of perioperative cardiovascular events in patients undergoing non-cardiac surgery.

## Methods

### Study design and population

This study is a prespecified analysis of the PANDA (Preoperative Coronary CT Angiography and Risk Evaluation in Non-cardiac Surgery Patients With Increased Risk of Cardiovascular Events) prospective observational trial, conducted at Gyeongsang National University Hospital, South Korea, between 2014 and 2016. The study design and primary findings have been reported previously [14]. The trial is registered at ClinicalTrials.gov (NCT02250963).

The PANDA trial enrolled patients undergoing intermediate- or high-risk non-cardiac surgery who had at least one clinical risk factor for perioperative cardiovascular events, as defined by the RCRI. Patients were excluded if they required emergency or low-risk procedures (e.g., endoscopic, superficial, or ambulatory surgery), had active cardiac conditions (e.g., recent MI, decompensated heart failure, significant valvular disease, or arrhythmias), had contraindications to CCTA or dobutamine stress echocardiography (DSE), or had an estimated glomerular filtration rate < 15 mL/min/1.73 m^2^.

**Fig. 1:**
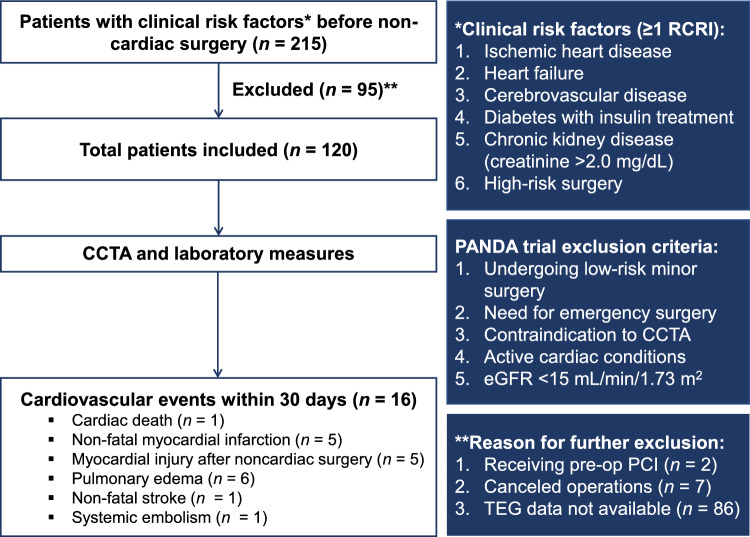
Flow diagram of the study. CKD = chronic kidney disease; CCTA = coronary computed tomography angiography; DM = diabetes mellitus; eGFR = estimated glomerular filtration rate; PCI = percutaneous coronary intervention; RCRI = Revised Cardiac Risk Index; TEG® = thromboelastography

Among 215 patients who underwent both CCTA and DSE, those who received prophylactic percutaneous coronary intervention (PCI; *n* = 2) or canceled surgery (*n* = 7) were excluded, leaving 206 patients. Of these, 120 had available TEG® data and were included in the present analysis (Fig. 1). The study was approved by the institutional review board, and all participants provided written informed consent.

### Clinical risk assessment

Baseline demographic, clinical, and surgical characteristics were collected. The RCRI was calculated based on six variables: ischemic heart disease, heart failure, cerebrovascular disease, insulin-dependent diabetes mellitus, renal insufficiency, and high-risk surgery [4].

### CCTA protocol and analysis

CCTA was performed as part of the preoperative evaluation at the discretion of the treating physician using a 64-slice multidetector scanner (Brilliance 64; Philips Medical Systems) [14]. Patients with resting heart rates > 65 beats per minute received oral propranolol (20–40 mg), and sublingual nitroglycerin (0.6 mg) was administered to all patients prior to image acquisition. An 80–100 mL bolus of non-ionic contrast was injected for coronary opacification, followed by a saline flush.

The coronary artery calcium score (CACS) was calculated using the Agatston method. All CCTA images were independently analyzed by two experienced cardiologists who were blinded to clinical outcomes. Coronary anatomy was categorized as normal (0% stenosis), non-significant (< 50% maximal stenosis), or significant (≥ 50% maximal stenosis). Multivessel disease was defined as the presence of significant stenosis in two or more major epicardial coronary arteries.

### Laboratory and TEG® measurements

Baseline biochemical assessments were performed using whole blood samples collected at hospital admission prior to surgery. N-terminal pro–B-type natriuretic peptide (NT-proBNP) was measured using an electrochemiluminescent immunoassay (Elecsys 2010 system, Roche Diagnostics, Mannheim, Germany). High-sensitivity C-reactive protein (hs-CRP) was assessed using an enzyme-linked immunosorbent assay on the UniCel® DxC 800 Synchron® system (Beckman Coulter, Brea, CA, USA). Fibrinogen and D-dimer levels were measured using the Clauss method on the STA-R Evolution® Hemostasis System (Diagnostica Stago, Paris, France). Additional laboratory tests, including blood chemistry, complete blood count, and lipid profile, were performed using standard automated assays. Postoperative troponin measurements were routinely obtained as part of perioperative care to assess myocardial injury.

TEG® was performed using the TEG® 5000 Hemostasis Analyzer System (Haemonetics Corp., Braintree, MA, USA) [12]. Citrated whole blood (3.2% trisodium citrate) was collected via antecubital venipuncture. For each assay, 500 μL of blood was activated with kaolin, and 340 μL of the mixture was transferred to a reaction cup with 20 μL of 200 mM calcium chloride to initiate clotting. TEG® parameters reflect the viscoelastic properties of clot formation and lysis. Reaction time (R) represents the time to initial fibrin formation (2 mm amplitude). K time reflects clot kinetics from R to 20 mm amplitude, while the alpha (α) angle indicates the rate of fibrin build-up and cross-linking. Maximum amplitude (MA), representing PFCS, reflects the combined contribution of platelet function and fibrinogen. Lysis at 30 min (LY_30_) denotes the percentage reduction in clot amplitude 30 min after MA and reflects fibrinolytic activity. We have previously demonstrated the association between elevated PFCS and adverse clinical outcomes in high-risk patients undergoing PCI [12, 13].

### Perioperative follow-up and outcome measures

All postoperative adverse events occurring within 30 days after surgery were independently adjudicated by two reviewers who were blinded to preoperative clinical data and assessed outcomes based on postoperative clinical information. The primary endpoint was a composite of perioperative cardiovascular events, including cardiovascular death, nonfatal MI, MINS, pulmonary edema, nonfatal stroke, or systemic embolism [14].

MI was defined in accordance with the universal definition, requiring an elevation in cardiac troponin with at least one of the following: symptoms of ischemia, new or presumed new significant ST-segment–T wave changes or new left bundle branch block, development of pathological Q waves on electrocardiography, or imaging evidence of new loss of viable myocardium or new regional wall motion abnormality [15]. MINS was defined as a postoperative elevation of cardiac troponin above the 99th percentile upper reference limit due to a presumed ischemic mechanism, in the absence of criteria for definite MI [16]. Symptoms in patients with elevated troponin levels were reviewed based on clinical documentation by the treating physicians and adjudicated by the study reviewers. Pulmonary edema was defined as new-onset pulmonary vascular congestion on postoperative chest radiography.

### Statistical analysis

Continuous variables were assessed for normality using the Kolmogorov–Smirnov test. Normally distributed variables are presented as mean ± standard deviation and were compared using the Student’s t-test, whereas skewed variables are presented as median (interquartile range [IQR]) and were compared using the Mann–Whitney U test. Categorical variables are expressed as counts and percentages and were compared using the chi-square or Fisher’s exact test, as appropriate.

Perioperative cardiovascular events were analyzed using logistic regression models to estimate odds ratios (ORs) and 95% confidence intervals (CIs). Candidate variables for multivariable analysis were selected a priori based on clinical relevance and grouped into predefined domains: clinical risk (RCRI), anatomical disease burden (presence of CAD on CCTA), and thrombogenicity (D-dimer and TEG®-PFCS). Receiver operating characteristic (ROC) curve analysis was performed to determine optimal cut-off values using the Youden index. Discriminative performance was assessed using the area under the curve (AUC), with comparisons performed using DeLong’s test. Incremental prognostic value was evaluated using the integrated discrimination improvement (IDI) and net reclassification improvement (NRI) with 95% CIs.

Prespecified nested logistic regression models were constructed to assess incremental value across domains: Model 1 (RCRI ≥ 2), Model 2 (Model 1 plus CAD), and Model 3 (Model 2 plus enhanced thrombogenicity). Given the limited number of events, a parsimonious modeling strategy was applied to minimize overfitting by restricting the number of variables included in each model. Model comparisons were performed using likelihood ratio tests. Model calibration was assessed using the Hosmer–Lemeshow goodness-of-fit test. Decision curve analysis was not performed due to the limited number of events, which may yield unstable estimates in small datasets.

A two-sided *P* value < 0.05 was considered statistically significant. All analyses were performed using SPSS (IBM Corp., Armonk, NY, USA) and R (R Foundation for Statistical Computing, Vienna, Austria).

## Results

### Baseline characteristics

Baseline clinical and laboratory characteristics of the 120 patients (mean age 69 ± 8 years; 57.5% male) are summarized according to the occurrence of postoperative cardiovascular events (Tables 1 and 2). Hypertension (69.2%) and diabetes mellitus (53.3%) were the most common comorbidities. Most patients had an RCRI score of 1 (60.8%) or 2 (30.8%), and 16.7% underwent high-risk surgery.

**Table 1:** Baseline characteristics according to postoperative cardiovascular events

Variables	Total (*n* = 120)	MACE (*n* = 16)	No MACE (*n* = 104)	*P-*value
RCRI, n (%)				0.012
1	73 (60.8)	6 (37.5)	67 (64.4)	
2	37 (30.8)	6 (37.5)	31 (29.8)	
3	9 (7.5)	4 (25.0)	5 (4.8)	
4	1 (0.8)	0 (0)	1 (1.0)	
Type of surgery, n (%)				0.339
Intermediate-risk	100 (83.3)	12 (75.0)	88 (84.6)	
High-risk	20 (16.7)	4 (20.0)	16 (15.4)	
Age, years	69 ± 8	73 ± 9	68 ± 7	0.051
Male sex, n (%)	69 (57.5)	11 (68.8)	58 (55.8)	0.328
Body mass index, kg/m^2^	23.2 ± 3.2	23.0 ± 2.5	23.3 ± 3.3	0.793
Risk factor or past medical history, n (%)				
Diabetes mellitus	64 (53.3)	10 (62.5)	54 (51.9)	0.430
Hypertension	83 (69.2)	13 (81.3)	70 (67.3)	0.385
Hypercholesterolemia	28 (23.3)	5 (31.3)	23 (22.1)	0.525
Smoking	27 (22.5)	5 (31.3)	22 (21.2)	0.352
Chronic kidney disease	22 (18.3)	5 (31.3)	17 (16.3)	0.170
Congestive heart failure	10 (8.3)	2 (12.5)	8 (7.7)	0.621
Peripheral arterial disease	8 (6.7)	1 (6.3)	7 (6.7)	1.000
COPD	12 (10)	0 (0)	12 (11.5)	0.364
Previous PCI	13 (10.8)	3 (18.8)	10 (9.6)	0.379
Previous MI	6 (5.0)	1 (6.3)	5 (4.8)	0.585
Previous stroke	32 (26.7)	6 (37.5)	26 (25.0)	0.363
Concomitant medications, n (%)				
Statin	36 (30.0)	7 (43.8)	29 (27.9)	0.243
Calcium channel blocker	51 (42.5)	9 (56.3)	42 (40.4)	0.232
Angiotensin blockade	58 (48.3)	11 (68.8)	47 (45.2)	0.079
Beta blocker	21 (17.5)	3 (18.8)	18 (17.3)	1.000
Nitrate	19 (15.8)	2 (12.5)	17 (16.3)	1.000
Diuretics	17 (14.2)	4 (25)	13 (12.5)	0.241
Proton pump inhibitor	5 (4.2)	1 (6.3)	4 (3.8)	0.517

COPD = chronic obstructive pulmonary disease; MACE = major adverse cardiovascular event; MI = myocardial infarction; PCI = percutaneous coronary intervention; RCRI = Revised Cardiac Risk Index

**Table 2:** Laboratory measurements according to postoperative cardiovascular events

Variables	Total (*n* = 120)	MACE (*n* = 16)	No MACE (*n* = 104)	*P*-value
CACS	107.2 (8.4–493.6)	234.3 (37.1–889.5)	102.6 (3.6–410.0)	0.150
Number of diseased coronary vessels, n (%)				0.001
0	78 (65.0)	5 (31.3)	73 (70.2)	
1	23 (19.2)	4 (25)	19 (18.3)	
2	15 (12.5)	6 (37.5)	9 (8.7)	
3	4 (3.3)	1 (6.3)	3 (2.9)	
WBC count, × 10^3^/mm^3^	7.4 ± 2.7	7.2 ± 2.2	74.2 ± 27.8	0.765
Hemoglobin, g/dL	11.8 ± 1.9	11.5 ± 1.8	11.9 ± 1.9	0.465
Platelet count, × 10^3^/mm^3^	240 ± 97	250 ± 113	239 ± 95	0.659
HbA1c, %	7.2 ± 2.1	6.7 ± 1.4	7.3 ± 2.1	0.465
Creatinine, mg/dL	0.8 (0.6–1.4)	0.8 (0.7–1.6)	0.8 (0.6–1.4)	0.313
BUN, mg/dL	16.6 ± 7.7	20.6 ± 11.8	16.0 ± 6.8	0.142
Total cholesterol, mg/dL	157 ± 36	143 ± 34	160 ± 36	0.078
Blood glucose, mg/dL	168.5 ± 87.8	151.8 ± 65.6	171.0 ± 90.7	0.417
Total bilirubin, mg/dL	0.55 ± 0.39	0.61 ± 0.27	0.54 ± 0.40	0.454
Total protein, g/dL	6.7 ± 0.7	6.6 ± 0.9	6.7 ± 0.7	0.514
Albumin, g/dL	3.8 ± 0.6	3.8 ± 0.6	3.5 ± 0.6	0.069
NT-proBNP, pg/mL	171.1 (46.0–429.3)	229.6 (59.8–2091.5)	171.1 (42.8–405.3)	0.398
Hemostatic measurement				
Fibrinogen, mg/dL	385 ± 111	422 ± 138	380 ± 106	0.158
D-dimer, μg/mL	1.5 (0.6–2.2)	2.3 (1.1–3.3)	1.3 (0.6–2.0)	0.022
PT, sec	14.6 ± 10.8	13.8 ± 1.0	14.7 ± 11.6	0.752
aPTT, sec	38.8 ± 13.7	41.7 ± 9.1	38.3 ± 14.3	0.365
TEG® indices				
R, min	5.7 ± 2.4	5.3 ± 1.6	5.8 ± 2.5	0.472
K, min	1.2 (0.9–1.6)	1.0 (0.8–1.2)	1.2 (0.9–1.7)	0.046
Alpha angle, degrees	68.8 ± 10.2	70.1 ± 10.7	68.6 ± 10.2	0.579
PFCS, mm	66.3 ± 9.0	69.9 ± 7.5	65.8 ± 9.1	0.084
LY_30_, %	0.1 (0–1.3)	0.3 (0.1–2.3)	0 (0–1.3)	0.085

aPTT = activated partial thromboplastin time; BUN = blood urea nitrogen; CACS = coronary artery calcium score; HbA1c = Hemoglobin A1c; K = clot kinetics; LY30 = clot lysis at 30 min; MACE = major adverse cardiovascular event; NT-proBNP = N-terminal pro–B-type natriuretic peptide; PFCS = platelet–fibrin clot strength; PT = prothrombin time; R = reaction time; TEG® = thromboelastography; WBC = white blood cell

During 30-day follow-up, 16 patients (13.3%) experience major adverse cardiovascular events (MACE), including 1 cardiovascular death, 5 MIs, 5 cases of MINS, 6 cases of pulmonary edema, 1 stroke, and 1 systemic embolism. Patients with MACE had higher RCRI scores than those without MACE (*P* = 0.012). Other baseline risk factors, comorbidities, and medications were comparable between groups.

CACS did not differ significantly between groups (234.3 [37.1–889.5] vs. 102.6 [3.6–410.0]; *P* = 0.150), whereas CAD was more prevalent in patients with MACE (68.7% vs. 29.8%; *P* = 0.001). D-dimer levels were also higher in patients with MACE (2.3 [1.1–3.3] vs. 1.3 [0.6–2.0] μg/mL; *P* = 0.022). Total cholesterol, albumin, and TEG®-PFCS showed modest, non-significant associations with postoperative MACE.

### Determinants of postoperative cardiovascular events

In multivariable analysis (Table 3), the presence of CAD, D-dimer, and TEG®-PFCS remained independently associated with postoperative MACE. CAD detected by CCTA was associated with an approximately fivefold increased risk (OR, 5.11; 95% CI, 1.49–17.53; *P* = 0.009). Among laboratory parameters, both D-dimer (per 1 μg/mL increase: OR, 1.22; 95% CI, 1.02–1.47; *P* = 0.030) and TEG®-PFCS (per 1 mm increase: OR, 1.10; 95% CI, 1.01–1.20; *P* = 0.027) were independently associated with MACE.

**Table 3:** Predictors of postoperative cardiovascular events

Variable	Univariate analysis			Multivariate analysis		
	OR	95% CI	*P*-value	OR	95% CI	*P*-value
RCRI ≥ 2	3.02	1.02–8.97	0.047	–	–	–
Age, per 1-year increase	1.08	1.00–1.16	0.055	–	–	–
Use of angiotensin blockade	0.38	0.12–1.16	0.087	–	–	–
Presence of CAD	5.18	1.66–16.16	0.005	5.11	1.49–17.53	0.009
Total cholesterol, per 1 mg/dL increase	0.99	0.97–1.00	0.077	–	–	–
Albumin, per 1 g/dL increase	0.45	0.18–1.08	0.074	–	–	–
NT-proBNP, per 1 pg/mL increase	1.00	1.00–1.00	0.056	–	–	–
D-dimer, per 1 μg/mL increase	1.16	1.00–1.35	0.054	1.22	1.02–1.47	0.030
PFCS (TEG®), per 1 mm increase	1.06	0.99–1.14	0.084	1.10	1.01–1.20	0.027

CAD = coronary artery disease; CI = confidence interval; HR = hazard ratio; NT-proBNP = N-terminal pro–B-type natriuretic peptide; PFCS = platelet–fibrin clot strength; RCRI = Revised Cardiac Risk Index; TEG® = thromboelastography

**Fig. 2:**
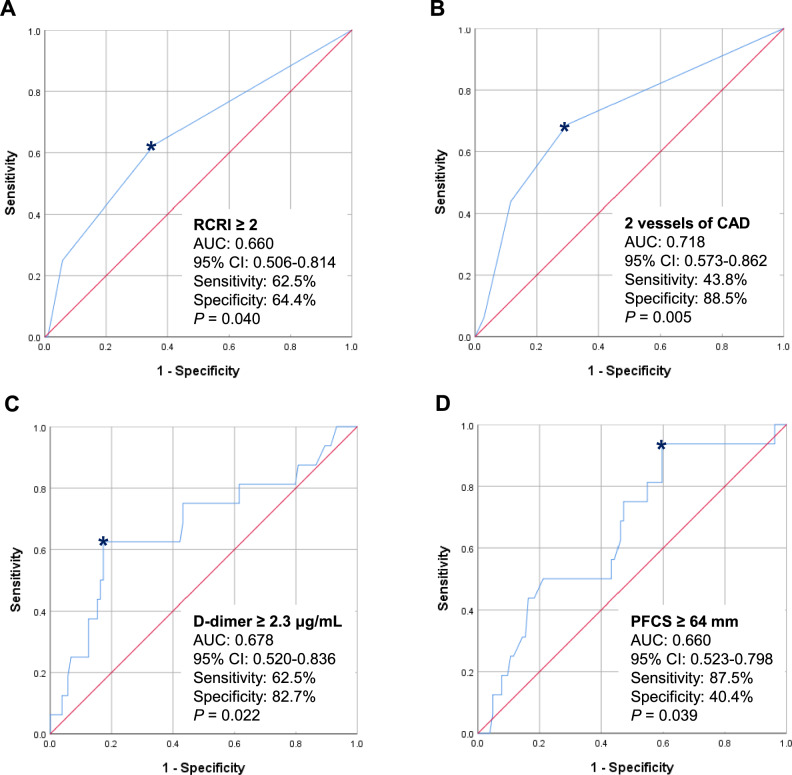
Receiver operating characteristic (ROC) curves for predicting perioperative cardiovascular events. (**a**) Predictive performance of the RCRI. (**b**) Number of coronary vessels with significant stenosis on CCTA. (**c**) D-dimer levels as a continuous variable. (**d**) PFCS, represented by maximum amplitude (MA) on TEG®. AUC = area under the curve; CAD = coronary artery disease; CCTA = coronary computed tomography angiography; CI = confidence interval; PFCS = platelet–fibrin clot strength; RCRI = Revised Cardiac Risk Index

ROC analysis identified optimal thresholds for predicting postoperative MACE. An RCRI score ≥ 2 and ≥ 2-vessel CAD on CCTA provided the best balance of sensitivity and specificity (Fig. 2A-B). A D-dimer threshold ≥ 2.3 µg/mL yielded 62.5% sensitivity and 82.7% specificity (AUC, 0.678; 95% CI, 0.520—0.836; *P* = 0.022) (Fig. 2C), while a TEG®-PFCS threshold ≥ 64 mm showed similar discrimination (AUC, 0.660; 95% CI, 0.5230–0.798; *P* = 0.039) (Fig. 2D).

When combined, a thrombogenicity phenotype (TEG®-PFCS ≥ 64 mm and D-dimer ≥ 2.3 µg/mL) was more frequent in patients with MACE than in those without (56.3% vs. 14.4%), and was associated with a nearly fourfold higher risk of events (OR, 3.90; 95% CI, 1.46–10.40; *P* = 0.0065).

### Predictive models integrating clinical, anatomical, and hemostatic profiles

**Fig. 3:**
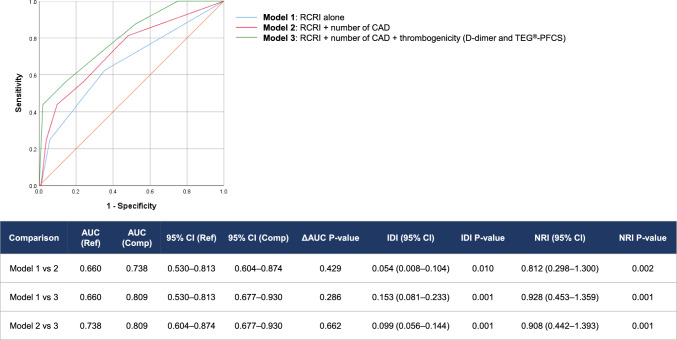
Prognostic value of coronary lesion and thrombogenicity profile for 30-day postoperative MACE. AUC = area under the curve; CAD = coronary artery disease; IDI = integrated discrimination improvement; MACE = major adverse cardiovascular events; NRI = net reclassification improvement; PFCS = platelet–fibrin clot strength; RCRI = Revised Cardiac Risk Index; TEG® = thromboelastography

Model 1 included the RCRI alone, Model 2 added CAD burden on CCTA, and Model 3 further incorporated thrombogenicity markers (D-dimer and TEG®-PFCS). Discrimination increased numerically across the models, with AUC values of 0.660 for Model 1 (95% CI, 0.530–0.813), 0.738 for Model 2 (95% CI, 0.604–0.874), and 0.809 for Model 3 (95% CI, 0.677–0.930), although differences were not statistically significant. The final model significantly enhanced both discrimination (IDI, 0.153; 95% CI, 0.081–0.233; *P* = 0.001) and reclassification (NRI, 0.928; 95% CI, 0.453–1.359; *P* = 0.001) compared with the RCRI-only model (Fig. 3).

In nested logistic regression, adding CAD to RCRI ≥ 2 improved model fit (Model 1 vs. Model 2: LR χ^2^ = 5.878; *P* = 0.015), with further improvement after incorporating thrombogenicity markers (Model 2 vs. Model 3: LR χ^2^ = 9.990; *P* = 0.0016). In the fully adjusted model, both CAD (OR, 4.35; 95% CI, 1.19–15.93; *P* = 0.026) and enhanced thrombogenicity (OR, 6.96; 95% CI, 2.08–23.28; *P* = 0.0016) remained independent predictors of perioperative cardiovascular events (Supplementary Table 1). The final model showed good calibration (Hosmer–Lemeshow *P* = 0.294), indicating agreement between predicted and observed event rates.

**Fig. 4:**
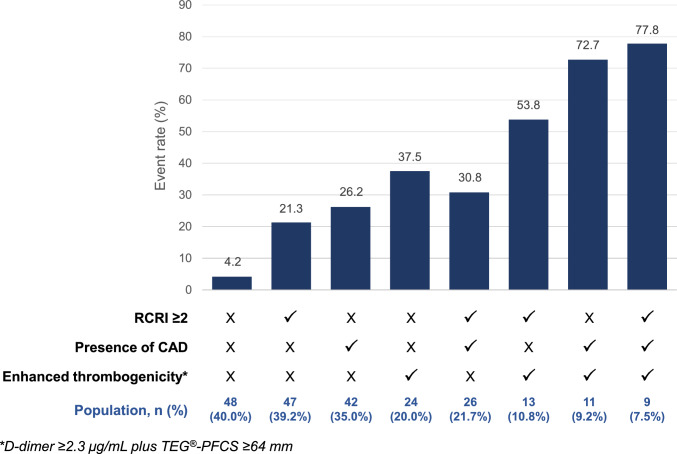
Perioperative cardiovascular incidence according to risk burden. CAD = coronary artery disease; PFCS = platelet–fibrin clot strength; RCRI = Revised Cardiac Risk Index; TEG® = thromboelastography

MACE incidence increased stepwise with the number of risk components (RCRI ≥ 2, CAD on CCTA, and enhanced thrombogenicity) (Fig. 4). Among these, enhanced thrombogenicity showed the strongest association with MACE. Event rates ranged from 4.2% in patients without any risk components to 77.8% in those with all three components.

## Discussion

To the best of our knowledge, this is among the first studies to evaluate the prognostic value of integrating thrombogenicity indices with CCTA for predicting cardiovascular complications after non-cardiac surgery. In this analysis, the RCRI showed modest discriminative performance. The addition of CCTA and thrombogenicity markers resulted in numerical improvements in model performance and enhanced risk reclassification. Conceptually, combining assessments of coronary anatomy (“vulnerable vessels”) and hemostatic profiles (“vulnerable blood”) may provide a more comprehensive evaluation of perioperative risk, but this approach remains exploratory and requires validation in larger studies.

### Prognostic role of preoperative biomarkers

Current guidelines support the use of biomarkers for perioperative risk assessment. The 2022 European Society of Cardiology (ESC) guideline recommend preoperative high-sensitivity cardiac troponin (hs-cTn) and suggest natriuretic peptide testing (BNP/NT-proBNP) in selected high-risk patients undergoing intermediate- or high-risk non-cardiac surgery [3]. Similarly, the 2024 American College of Cardiology/American Heart Association (ACC/AHA) guideline support natriuretic peptide testing (Class IIa) and provide a weaker recommendation for troponins (Class IIb) [17]. However, their clinical utility is limited by confounding factors (e.g., age and renal dysfunction), variability in assay thresholds, and the lack of evidence that biomarker-guided management improves outcomes. In addition, their use is restricted to selected high-risk populations and may lead to downstream testing, surgical delays, and challenges in interpreting baseline elevations.

Perioperative cardiovascular events arise from overlapping mechanisms, including myocardial oxygen supply–demand imbalance (leading to MINS or type 2 MI) and inflammation-driven thrombogenicity, which may trigger plaque disruption, coronary thrombosis, or microvascular injury [18, 19]. These mechanisms underscore the importance of assessing the coagulation–fibrinolysis axis, as both thrombotic and bleeding complications are linked to adverse outcomes [20, 21]. In this context, our findings support the potential prognostic value of thrombogenicity-related biomarkers, including TEG®-derived PFCS and D-dimer, in capturing perioperative thrombotic risk. However, thrombogenicity reflects a complex and dynamic interplay between platelet function, coagulation pathways, thrombin generation, and fibrinolysis, making its assessment inherently challenging [11].

TEG®, an ex vivo viscoelastic assay, provides a global, real-time assessment of coagulation kinetics, clot strength, and fibrinolysis [10]. TEG®-PFCS integrates multiple pathophysiological processes, including platelet function, fibrinogen activity, and inflammatory status, thereby serving as a composite marker of thrombotic potential [22]. Elevated PFCS may reflect a hypercoagulable state, which is common in the perioperative period and particularly relevant in patients with underlying CAD [13, 22, 23]. D-dimer reflects ongoing coagulation and fibrinolysis and is widely used in the evaluation of thrombotic disorders [24]. Although nonspecific and influenced by factors such as surgery or infection, elevated D-dimer levels have been associated with increased thrombotic risk and may provide prognostic value in the perioperative setting [25].

Importantly, our findings suggest that an integrated thrombogenicity profile combining D-dimer and TEG®-PFCS may offer complementary information beyond conventional clinical risk scores. Age-adjusted D-dimer thresholds are commonly used in the diagnostic evaluation of venous thromboembolism to improve specificity while maintaining sensitivity [26]. In the present study, however, D-dimer was not used as a diagnostic test but as a marker of perioperative thrombogenicity. Therefore, a study-specific cut-off derived from ROC analysis was applied to optimize prediction of perioperative cardiovascular events.

### Risk stratification using CCTA

CCTA enables detailed anatomical assessment of CAD and is recommended primarily for patients with low-to-intermediate clinical likelihood of CAD [27]. However, its role in perioperative risk stratification has gained increasing interest, with prior studies demonstrating both diagnostic and prognostic utility in patients undergoing non-cardiac surgery [6, 28–30]. In line with these data, our study found that the presence of significant CAD—particularly multivessel disease—was independently associated with perioperative cardiovascular events.

Despite this association, adding CCTA to clinical risk models did not significantly improve overall discrimination. This may reflect overlap between clinical risk factors captured by the RCRI and underlying anatomical disease, as well as limited statistical power due to the small number of events. Importantly, this finding should not be interpreted as diminishing the value of CCTA, but rather highlights that anatomical and clinical risk assessments provide related, and potentially complementary, information.

CACS, while well established for long-term risk prediction, showed limited incremental value in our analysis. This may be due to its inability to characterize luminal stenosis or plaque features that are more relevant to short-term perioperative risk [31]. In addition, our study utilized 64-slice CCTA; contemporary technologies allow more detailed plaque characterization (e.g., low-attenuation plaque, positive remodeling), which may enhance predictive performance and limit the generalizability of our findings.

From a clinical perspective, CCTA should be applied within an individualized, risk-adapted framework, balancing its diagnostic value against potential risks such as radiation exposure and contrast-associated nephropathy [5]. Rather than replacing established clinical risk scores, CCTA may serve as a complementary tool—RCRI for initial risk stratification and CCTA for more refined anatomical assessment in selected patients.

Importantly, improved anatomical risk detection does not necessarily translate into improved clinical outcomes. Prior studies have suggested that CCTA may lead to overestimation of perioperative risk, potentially resulting in unnecessary downstream testing or delays in surgery [6]. In addition, the Coronary Artery Revascularization Prophylaxis trial demonstrated that routine preoperative coronary revascularization does not reduce perioperative death or MI and may delay surgical procedures [32]. Therefore, our findings should not be interpreted as supporting routine use of CCTA to guide revascularization, but suggest that integrating anatomical information with clinical and thrombogenicity profiles may provide incremental insight into perioperative risk stratification.

### Multimodal risk stratification: bridging thrombogenicity and imaging

The addition of CAD burden and thrombogenicity markers to the RCRI was associated with improved overall model performance. Although multimodal models showed numerically higher AUC values compared with the RCRI alone, these differences were not statistically significant. This may reflect both partial overlap between predictors and limited statistical power given the small number of events.

In contrast, reclassification metrics (IDI and NRI) suggested improved risk stratification with the addition of anatomical and thrombogenicity information. These findings should be interpreted cautiously. The AUC (equivalent to the C-statistic in logistic regression models) primarily reflects a model’s ability to rank individuals by risk and is relatively insensitive to changes in absolute risk estimates unless these alter the ranking [33]. Reclassification metrics, in contrast, capture changes in predicted risk at the individual level and may therefore detect shifts not reflected in the AUC [34]. However, NRI and IDI have recognized limitations and should be considered complementary rather than definitive indicators of improved predictive performance.

Overall, integrating clinical, anatomical, and thrombogenicity information may provide additional insights into perioperative risk assessment. In this context, thrombogenicity markers such as TEG®-PFCS and D-dimer should be viewed primarily as risk stratification tools rather than therapeutic targets. Patients with both significant CAD and elevated thrombogenicity appeared more likely to be categorized as higher risk. Accordingly, such patients may warrant closer monitoring and optimized perioperative management, rather than routine escalation of antithrombotic therapy.

An important consideration is the cost and resource implications of this multimodal approach. Unlike the RCRI, which requires no additional testing, this strategy incorporates imaging (CCTA) and specialized assays (TEG® and D-dimer), potentially increasing healthcare costs and limiting accessibility. Formal cost-effectiveness analyses and validation in larger cohorts are needed.

### Medical therapy to prevent postoperative cardiovascular events

Optimization of baseline cardiovascular risk factors and continuation of guideline-directed medical therapy remain the cornerstone of perioperative management [3, 17]. Statins provide well-established long-term cardiovascular benefits through lipid-lowering and pleiotropic anti-inflammatory effects and are generally recommended to be continued in the perioperative period. However, evidence supporting routine initiation of statins immediately before non-cardiac surgery remains limited, and their benefit is primarily derived from chronic use rather than short-term perioperative intervention [35].

Similarly, antiplatelet therapy such as aspirin should be individualized based on established indications, balancing ischemic and bleeding risks. In the Perioperative Ischemic Evaluation 2 (POISE-2) trial, which enrolled patients undergoing non-cardiac surgery, perioperative aspirin did not reduce major cardiovascular events but increased bleeding risk [36]. Direct oral anticoagulants are primarily used for postoperative thromboprophylaxis in selected surgical populations and are not routinely indicated for prevention of arterial cardiovascular events in this setting [37].

### Limitations

Several limitations should be acknowledged. First, the single-center design, relatively small sample size, and limited number of events may restrict generalizability and model stability. Larger multicenter prospective studies are required for validation. For the same reason, advanced model evaluation methods such as decision curve analysis were not performed, as estimates may be unreliable in small datasets. Second, potential selection bias should be considered. Patients with contraindications to iodinated contrast (e.g., prior hypersensitivity or advanced chronic kidney disease) were excluded from CCTA, limiting applicability to this high-risk subgroup. In addition, preoperative CCTA and biomarker results may have influenced subsequent management, including the implementation of preventive strategies. Third, measurement-related limitations exist. Variability in the timing of CCTA and laboratory assessments may have affected biomarker levels, and serial measurements of thrombogenicity were not performed. Therefore, dynamic changes over time and their potential impact on postoperative cardiovascular risk could not be evaluated. Finally, outcome-related considerations should be noted. The inclusion of MINS may have increased event rates by capturing biomarker-defined, often subclinical events. Moreover, given the limited number of events and the lack of systematic classification of MI subtypes, further stratified analyses were not feasible.

## Conclusions

In this prospective analysis, integrating anatomical and hemostatic assessments provided incremental value for perioperative cardiovascular risk stratification. CAD detected by CCTA and an enhanced thrombogenicity profile (D-dimer and TEG®-PFCS) were independently associated with postoperative cardiovascular events. These findings support a multimodal approach to perioperative risk assessment, although validation in larger, external cohorts is required before routine clinical implementation.

## Supplementary Information

Below is the link to the electronic supplementary material.

Supplementary file1 (DOCX 15 KB)

## Data Availability

The data that support the findings of this study are available from the corresponding author upon reasonable request.

## Abbreviations

CACS: Coronary artery calcium score
CAD: Coronary artery disease
CCTA: Coronary computed tomography angiography
MI: Myocardial infarction
MINS: Myocardial injury after non-cardiac surgery
NT-proBNP: N-terminal pro–B-type natriuretic peptide
PCI: Percutaneous coronary intervention
PFCS: Platelet–fibrin clot strength
RCRI: Revised Cardiac Risk Index
TEG®: Thromboelastography
