# Supplementary material for: Association of thrombogenicity indices with perioperative cardiovascular events after non-cardiac surgery: a prespecified analysis of the PANDA study
Source: J Thromb Thrombolysis. 2026 May 18;59(6):1349–61. doi: 10.1007/s11239-026-03299-z (PMC13447407; doi:10.1007/s11239-026-03299-z)
Supplement: Supplementary file 1 [file 11239_2026_3299_MOESM1_ESM.docx]

**Table S1. Nested logistic regression models for prediction of perioperative cardiovascular events**

| **Variable** | **Model 1 (clinical)**  **OR (95% CI)** | **P value** | **Model 2 (clinical + anatomical)**  **OR (95% CI)** | **P value** | **Model 3 (clinical + anatomical + thrombogenicity)**  **OR (95% CI)** | **P value** |
| --- | --- | --- | --- | --- | --- | --- |
| **RCRI ≥2** | 3.02 (1.02–8.97) | 0.047 | 1.91 (0.59–6.17) | 0.277 | 1.43 (0.40–5.16) | 0.580 |
| **CAD presence** | — | — | 4.19 (1.27–13.87) | 0.019 | 4.35 (1.19–15.93) | 0.026 |
| **Enhanced thrombogenicity profile*** | — | — | — | — | 6.96 (2.08–23.28) | 0.0016 |

*Enhanced thrombogenicity profile was defined as D-dimer ≥2.3 μg/mL and TEG-PFCS ≥64 mm.

Nested model comparisons using likelihood ratio tests showed significant improvement in model fit with sequential addition of predictors: Model 1 vs Model 2 (LR χ² = 5.878, df = 1, P = 0.015), Model 2 vs Model 3 (LR χ² = 9.990, df = 1, P = 0.0016), and Model 1 vs Model 3 (LR χ² = 15.867, df = 2, P < 0.001). Abbreviations: RCRI, Revised Cardiac Risk Index; CAD, coronary artery disease; TEG, thromboelastography; PFCS, platelet–fibrin clot strength.

| **Comparison** | **LR χ²** | **df** | **P value** |
| --- | --- | --- | --- |
| Model 1 vs Model 2 | 5.878 | 1 | 0.015 |
| Model 2 vs Model 3 | 9.990 | 1 | 0.0016 |
| Model 1 vs Model 3 | 15.867 | 2 | 0.00036 |
